# Arnold-Chiari Malformation Type I Presenting With Bilateral Scapular Winging

**DOI:** 10.7759/cureus.76330

**Published:** 2024-12-24

**Authors:** Aljomon D Leaño, Bonifacio C Pedregosa, Steven G Villaraza, Jose C Navarro

**Affiliations:** 1 Department of Neurology, Jose R. Reyes Memorial Medical Center, Manila, PHL

**Keywords:** arnold-chiari malformations, congenital neurological disorders, neurology, scapular winging, syringomyelia

## Abstract

Bilateral scapular winging is a rare and atypical manifestation of Arnold-Chiari malformation type 1 (ACM1). We report a case of ACM with extensive syrinx formation, presenting with progressive bilateral proximal upper extremity weakness, bilateral scapular winging, and segmental hypesthesia and reduced thermesthesia over the bilateral C2-C8 dermatomal levels. The patient was successfully treated with surgical decompression and syringosubarachnoid shunting. This unusual presentation of ACM1 emphasizes the need to consider central nervous system pathologies in the differential diagnosis for bilateral scapular winging.

## Introduction

Scapular winging is caused by weakness of the serratus anterior, rhomboid, or trapezius muscles [1]. Although typically due to trauma, myopathies, peripheral neuropathies, or surgical complications, this neurologic sign may result from an underlying central nervous system disorder [1].

We report the case of a patient with bilateral scapular winging caused by Arnold-Chiari malformation type I (ACM1) with extensive syrinx formation. ACM1 is a congenital hindbrain herniation syndrome characterized by ectopia of the cerebellar tonsils into the spinal canal without caudal displacement of the medulla and is often complicated by the development of syringohydromyelia [2,3].

Bilateral scapular winging is an exceedingly rare manifestation of ACM1, with only one other documented case in the literature [4].

## Case presentation

A 33-year-old male presented with a two-year history of progressively worsening proximal weakness of the bilateral upper extremities. He had difficulty with overhead activities but had no impairment in skilled manual tasks involving his hands. There were no other symptoms reported. He was previously well with no known comorbidities or prior trauma. A review of his family history was non-contributory.

Neurologic examination revealed bilateral scapular winging (serratus anterior type), mild paresis of the bilateral triceps and serratus anterior muscles (graded 4/5 on the Medical Research Council (MRC) scale for muscle strength), attenuation of the bilateral triceps deep tendon reflexes, and absence of long tract signs. Hypoalgesia and hypothermesthesia were noted over the bilateral C2 to C8 dermatomal levels. The patient had intact cognition, normal cranial nerve function, and no other focal neurologic deficits. His general physical examination was unremarkable.

**Figure 1:**
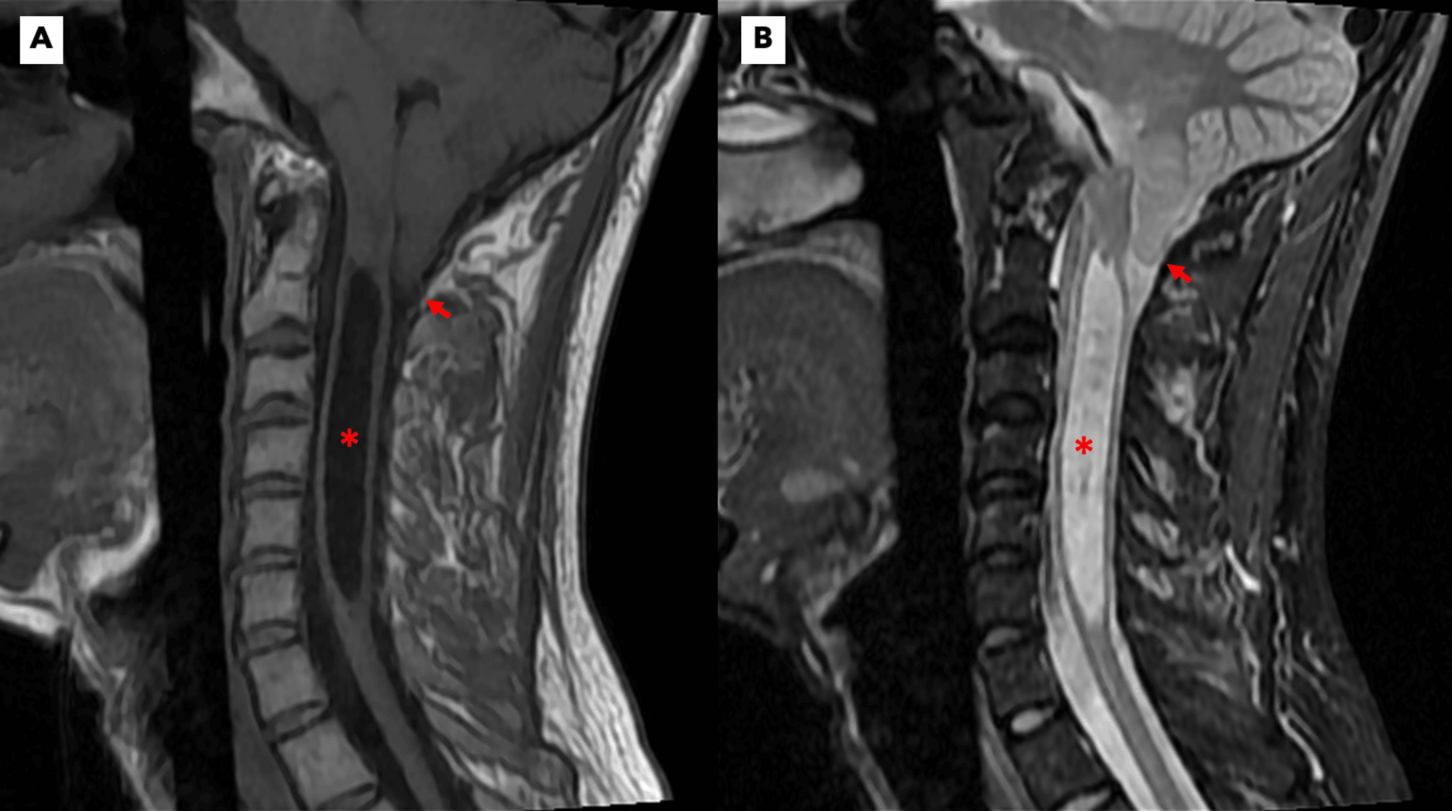
Pre-operative cervical MRI findings Sagittal T1 (A) and T2 (B) weighted MRI demonstrate herniation of the cerebellar tonsils through the foramen magnum (arrows). A large, centrally located syrinx (stars) is seen spanning C1 to C6, causing expansion of the spinal cord. These findings are consistent with ACM1. ACM1: Arnold-Chiari malformation type I; MRI: magnetic resonance imaging

Cervical magnetic resonance imaging (MRI) (Figure 1) demonstrated herniation of the cerebellar tonsils, along with an extensive, centrally located syrinx from C1 to C6. The findings are consistent with ACM1.

**Figure 2:**
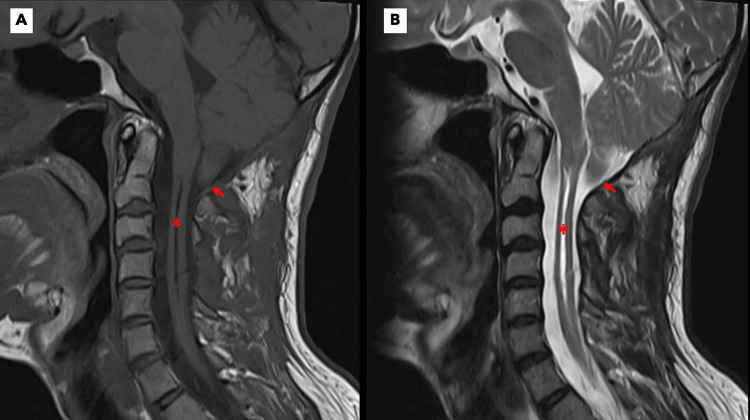
Post-operative cervical MRI findings Sagittal T1 (A) and T2 (B) weighted MRI demonstrate a marked reduction in the volume of the previously noted syrinx (stars), with the persistence of tonsillar herniation (arrows). MRI: magnetic resonance imaging

The patient eventually underwent decompressive suboccipital craniectomy and C1 laminectomy with duraplasty and insertion of a syringosubarachnoid shunt. Post-operatively, there was complete resolution of sensory deficits with moderate improvement of the bilateral triceps and anterior serratus paresis (now graded 4+/5 on the MRC scale); scapular winging was still evident. Repeat cervical MR imaging (Figure 2) revealed interval regression in the size of the syrinx with the persistence of tonsillar ectopia. On the three-month follow-up, the neurologic examination was unchanged.

## Discussion

ACM, first described by Hans Chiari in 1891, represents a range of congenital hindbrain herniation syndromes [2]. Type I malformations are characterized by the ectopia of the cerebellar tonsils through the foramen magnum unaccompanied by any caudal displacement of the medulla and are typically the mildest variant, often presenting in adulthood at an average age of 30-35 years [3]. Symptomatic ACM1 is estimated to affect approximately 0.1% of the general population, with concurrent syringomyelia occurring in 37% to 75% of these cases [3,5].

The exact etiopathogenesis of ACM1 remains unclear. While no single theory fully explains the entire spectrum of observed abnormalities, it is likely that ACM1 arises from a dynamic interplay of various factors including neural hindbrain dysgenesis, osseous dysplasia, and alterations in cerebrospinal fluid hydrodynamics [2,3,6-9]. The disruption of normal CSF flow by structural anomalies within the posterior fossa and craniocervical junction is hypothesized to contribute to syrinx formation [10]. Genetic factors may also play a role, as evidenced by familial cases of ACM1 [11]. Ultimately, it is the interaction of these factors that likely determines the onset, progression, and variability in clinical manifestations.

The clinical heterogeneity of ACM1 reflects the diverse neuroanatomic pathways potentially affected by the underlying structural abnormalities. Manifestations are determined primarily by the degree of tonsillar herniation, the presence and extent of any associated syrinx, and whether brainstem compression or obstructive hydrocephalus is present [3]. In our patient, bilateral segmental sensory deficits and scapular winging were likely caused by the central cord lesion disrupting the anterior white commissure and the bilateral motor nuclei, which supply the serratus anterior muscles via the long thoracic nerves, respectively.

Treatment of ACM1 is primarily surgical, with the primary goal of relieving spinal cord compression and restoring cerebrospinal fluid flow; concurrent syringomyelia often necessitates shunting [3]. Prognosis and long-term response to surgical interventions are highly variable, with approximately 25% of patients who initially responded favorably eventually deteriorating [12,13]. Given the limited understanding of the natural history of this condition, the true benefits of surgery over its natural course remain unclear. The presence of a concurrent syrinx is associated with a poorer prognosis [3].

## Conclusions

We report a rare case of ACM1 presenting with bilateral scapular winging, highlighting an unusual manifestation of ACM1 and emphasizing the need to consider central nervous system pathologies as part of the differential diagnosis for bilateral scapular winging.
